# Angulin proteins ILDR1 and ILDR2 regulate alternative pre-mRNA splicing through binding to splicing factors TRA2A, TRA2B, or SRSF1

**DOI:** 10.1038/s41598-017-07530-z

**Published:** 2017-08-07

**Authors:** Yueyue Liu, Hongyun Nie, Chengcheng Liu, Xiaoyan Zhai, Qing Sang, Yanfei Wang, Deli Shi, Lei Wang, Zhigang Xu

**Affiliations:** 1 Shandong Provincial Key Laboratory of Animal Cells and Developmental Biology, Shandong University School of Life Sciences, Jinan, Shandong 250100 China; 2 State Key Laboratory of Genetic Engineering and MOE Key Laboratory of Contemporary Anthropology, School of Life Sciences, Fudan University, Shanghai, 200032 China; 3 Laboratoire de Biologie du Développement, Institut de Biologie Paris-Seine, Sorbonne Universités, Paris, France

## Abstract

Angulin proteins are a group of evolutionally conserved type I transmembrane proteins that contain an extracellular Ig-like domain. In mammals, three angulin proteins have been identified, namely immunoglobulin-like domain containing receptor 1 (ILDR1), immunoglobulin-like domain containing receptor 2 (ILDR2), and lipolysis-stimulated lipoprotein receptor (LSR). All three proteins have been shown to localize at tight junctions (TJs) and are important for TJ formation. Mutations in *ILDR1* gene have been shown to cause non-syndromic hearing loss (NSHL). In the present work, we show that ILDR1 binds to splicing factors TRA2A, TRA2B, and SRSF1, and translocates into the nuclei when the splicing factors are present. Moreover, ILDR1 affects alternative splicing of *Tubulin delta 1* (*TUBD1*), *IQ motif containing B1* (*IQCB1*), and *Protocadherin 19* (*Pcdh19*). Further investigation show that ILDR2, but not LSR, also binds to the splicing factors and regulates alternative splicing. When endogenous ILDR1 and ILDR2 expression is knockdown with siRNAs in cultured cells, alternative splicing of *TUBD1* and *IQCB1* is affected. In conclusion, we show here that angulin proteins ILDR1 and ILDR2 are involved in alternative pre-mRNA splicing via binding to splicing factors TRA2A, TRA2B, or SRSF1.

## Introduction

Immunoglobulin-like domain containing receptor 1 (ILDR1) is a putative type I transmembrane protein containing an immunoglobulin (Ig)-like extracellular N-terminal domain^1^. ILDR1 belongs to an evolutionally conserved protein family, angulin protein family, which also includes immunoglobulin-like domain-containing receptor 2 (ILDR2) and lipolysis-stimulated lipoprotein receptor (LSR)^2^. Mutations of *ILDR1* gene are responsible for autosomal recessive hearing impairment DFNB42^3^. *Ildr1* knockout mice show profound hearing loss accompanied with postnatal cochlear hair cell degeneration^4–6^. In mouse inner ear, *Ildr1* mRNA was detected in hair cells as well as supporting cells, and ILDR1 protein was shown to localize at tricellular tight junctions (tTJs)^2, 3^. Another tight junction protein, tricellulin, is also required for the structure and function of tTJs, and mutations of *tricellulin* gene cause autosomal recessive hearing impairment DFNB49^7^. ILDR1 recruits tricellulin to tTJs, whereas DFNB42-associated ILDR1 mutant protein fails to do so^2^.

Although ILDR1 is suggested to play an important role in regulating the integration of tTJs, it seems that this protein is also involved in functions other than tTJs in the inner ear. Consistent with this hypothesis, hair cell degeneration in *Ildr1* knockout mice is more severe than that in tricellulin mutant mice^4, 8^. Moreover, ILDR1 has been shown to mediate fat-stimulated cholecystokinin (CCK) secretion, raising the possibility that ILDR1 could act as a signaling receptor^9^. Nevertheless, the role of ILDR1 other than regulating tTJs largely remains unknown at present.

Alternative splicing is important for regulation of protein function and proteomic diversity. During alternative splicing, the exons of pre-mRNAs are spliced together in different arrangements to give rise to distinct mature mRNAs, which ultimately produces structurally and functionally distinct protein variants^10^. Alternative splicing deficits cause various types of disease including hearing loss^11, 12^. At present, two splicing factors have been identified to play important roles in the inner ear, namely SRRM4 and SFSWAP^13, 14^, both of which belong to SR protein family. SR proteins were first discovered in *Drosophila* when Sfswap, Tra, and Tra-2 were identified as splicing factors through genetic screens^15–17^. All SR proteins contain an arginine/serine-rich region, which was then referred as RS domain. Besides RS domain, typical SR proteins also contain a so-called RNA recognition motif (RRM), which provides RNA-binding specificity. Further studies revealed that SR proteins are phylogenetically conserved and structurally related proteins that are involved in both constitutive and alternative splicing^18, 19^.

Transformer 2 protein homolog alpha (TRA2A), transformer 2 protein homolog beta (TRA2B), and serine/arginine-rich splicing factor 1 (SRSF1) are also SR protein family members. TRA2A and TRA2B are mammalian homologs of fly Tra-2, and have been shown to play important roles in alternative splicing^20, 21^. SRSF1, originally named SF2 or ASF, is the first identified mammalian SR protein^22, 23^. Microarray and RNA sequencing have revealed that *Tra2a*, *Tra2b*, and *Srsf1* are expressed in the mouse inner ear, though their precise expression pattern and functional role in the inner ear remain elusive^24–26^. In the present work, we show that ILDR1 as well as its paralog ILDR2 could regulate alternative pre-mRNA splicing via binding to TRA2A, TRA2B, and SRSF1.

## Results

### Identification of TRA2A, TRA2B, and SRSF1 as ILDR1-binding partners

**Figure 1:**
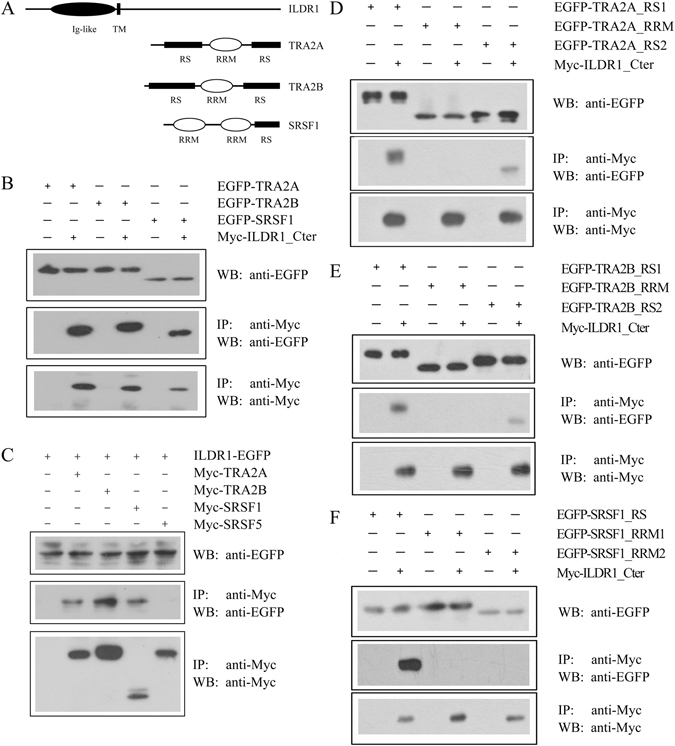
ILDR1 interacts with TRA2A, TRA2B, and SRSF1. (**A**) Schematic drawing of the domain structures of *Mus musculus* ILDR1, TRA2A, TRA2B, and SRSF1. (**B**) Western blots showing that EGFP-tagged TRA2A, TRA2B, and SRSF1 were co-immunoprecipitated with Myc-tagged ILDR1 cytoplasmic fragment. (**C**) Western blots showing that EGFP-tagged ILDR1 was co-immunoprecipitated with Myc-tagged TRA2A, TRA2B, SRSF1, but not SRSF5. (**D**) Western blots showing that EGFP-tagged TRA2A RS domain, but not RRM domain, was co-immunoprecipitated with Myc-tagged ILDR1 cytoplasmic fragment. (**E**) Western blots showing that EGFP-tagged TRA2B RS domain, but not RRM domain, was co-immunoprecipitated with Myc-tagged ILDR1 cytoplasmic fragment. (**F**) Western blots showing that EGFP-tagged SRSF1 RS domain, but not RRM domain, was co-immunoprecipitated with Myc-tagged ILDR1 cytoplasmic fragment. Expression vectors were transfected into HEK293T cells to express epitope-tagged proteins, and cell lysis were subject to immunoprecipitation. 5% of total protein was loaded as input. IP indicates antibody used for immunoprecipitation and WB indicates antibody used for detection. Uncropped blots are shown in Fig. S9.

In order to identify ILDR1-binding partners in the inner ear, we performed yeast two-hybrid screening of a chicken cochlear cDNA library using the C-terminal intracellular domain of chicken ILDR1 (228–553 aa) as bait. Among the positive clones identified, several clones encode for a group of splicing factors, including TRA2A, TRA2B, and SRSF1 (Table 1). TRA2A, TRA2B, and SRSF1 belong to SR protein family, which share one or two serine/arginine-rich domain (RS domain) as well as RNA recognition motif (RRM) (Fig. 1A). SR proteins have been shown to play important roles in constitutive as well as alternative pre-mRNA splicing^18^.

**Table 1:** Potential ILDR1-binding partners identified from yeast two-hybrid screening.

GenBank accession No.	Prey protein	Prey redundancy
NP_001006360	transformer-2 protein homolog alpha (TRA2A)	5
NP_990009	transformer-2 protein homolog beta (TRA2B)	3
NP_001107213	serine/arginine-rich splicing factor 1 (SRSF1)	2

We then performed co-immunoprecipitation (co-IP) experiments to confirm the interaction between ILDR1 and the splicing factors. ILDR1 is quite conserved in vertebrates. Chicken and mouse ILDR1 share 95% and 60% homology in the extracellular part and the C-terminal end, respectively. In the following work we focus on mouse proteins. The co-IP results showed that EGFP-tagged mouse TRA2A, TRA2B, or SRSF1 is co-immunoprecipitated with Myc-tagged ILDR1 cytoplasmic domain (Fig. 1B). Likewise, EGFP-tagged full-length ILDR1 could be co-immunoprecipitated with Myc-tagged TRA2A, TRA2B, or SRSF1 (Fig. 1C). As a control, another SR protein, SRSF5, was included in the experiment, and the results showed that SRSF5 is not co-immunoprecipitated with ILDR1, confirming the specific interaction between ILDR1 and TRA2A/TRA2B/SRSF1 (Fig. 1C). To further narrow down which domain(s) of TRA2A/TRA2B/SRSF1 is required for the interaction, we performed co-IP experiments with different domains of TRA2A/TRA2B/SRSF1. The results showed that the RS domain is responsible for the interaction with ILDR1 (Fig. 1D–F).

Three *ILDR1* splicing transcriptional variants have been identified, namely *ILDR1α*, *ILDR1α*, and *ILDR1β* ^1^. Full-length ILDR1 (ILDR1α) contains a di-leucine motif and a cysteine-rich region in the cytoplasmic part, and is usually simply referred as ILDR1 for convenience. Compared to ILDR1α, ILDR1α’ misses the di-leucine motif, whereas ILDR1β misses the transmembrane domain as well as the cysteine-rich region (Fig. S1A). Expression of *ILDR1α* and *ILDR1α’*, but not *ILDR1β*, was detected in mouse inner ear by RT-PCR experiment (data not shown). We then amplified the cDNA of *ILDR1α’* from mouse inner ear and performed co-IP experiment. The result showed that unlike ILDR1α, ILDR1α’ cytoplasmic domain was not co-immunoprecipitated with TRA2B, suggesting that the di-leucine motif is necessary for the interaction between ILDR1 and the splicing factors (Fig. S1B).

### ILDR1 translocates into the nuclei when TRA2A, TRA2B, or SRSF1 is present

**Figure 2:**
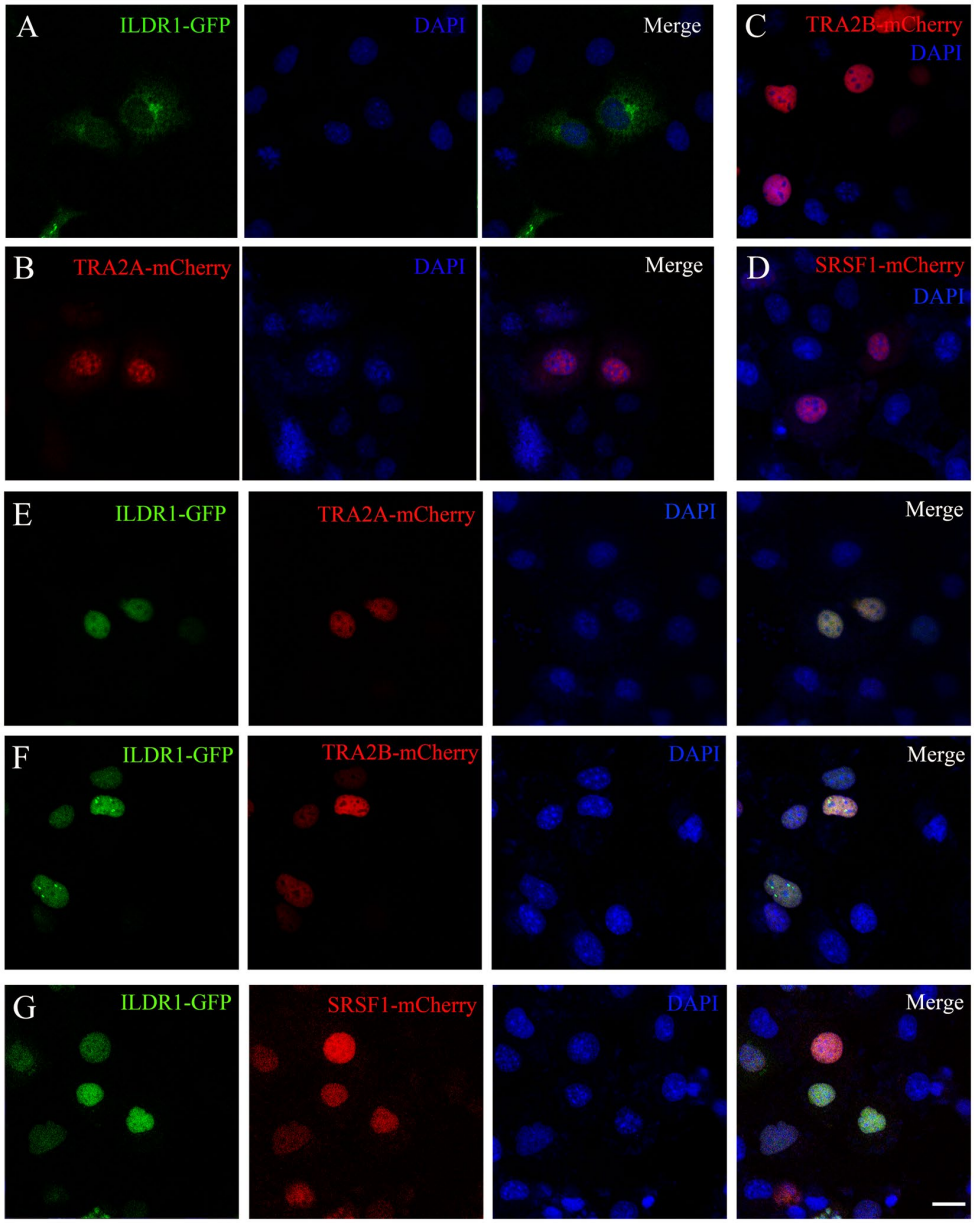
ILDR1 translocates into the nuclei when TRA2A, TRA2B, or SRSF1 is present. (**A**) ILDR1-GFP localizes in the cytoplasm. (**B–D**) TRA2A-mCherry, TRA2B-mCherry, and SRSF1-mCherry localize in the nuclei. (**E–G**) When cotransfected, ILDR1-GFP moves into the nuclei, colocalizing with TRA2A-mCherry, TRA2B-mCherry, or SRSF1-mCherry. Expression vectors were transfected into COS-7 cells to express epitope-tagged proteins. Nuclei were stained with DAPI. Scale bar: 20 μm.

Exogenous ILDR1 has been shown to localize in the cytoplasm of HEK293T cells^1^. Consistently, we found that ILDR1-GFP mainly localizes in the cytoplasm in COS-7 cells (Fig. 2A). In contrast, TRA2A-mCherry, TRA2B-mCherry, and SRSF1-mCherry localize exclusively in the nuclei (Fig. 2B–D). Interestingly, when cotransfected together with TRA2A-mCherry, TRA2B-mCherry, or SRSF1-mCherry, ILDR1-GFP translocates from the cytoplasm into the nuclei (Fig. 2E–G, Fig. S2). The recruitment of ILDR1 into the nuclei by these splicing factors suggests that ILDR1 might play a role in splicing regulation. Noticeably, ILDR1α’-GFP does not move into the nuclei when TRA2A/TRA2B/SRSF1 is present (Fig. S1C–F), consistent with the finding that ILDR1α’ does not interact with these splicing factors (Fig. S1B).

### *Tra2a*, *Tra2b*, and *Srsf1* are expressed in the mouse inner ear

Transcriptome analysis has suggested that *Tra2a*, *Tra2*b, and *Srsf1* are expressed in the mouse inner ear (SHIELD; https://shield.hms.harvard.edu)^25, 26^. To examine their expression pattern in the cochlea, *in situ* hybridization was performed using whole-mount mouse cochlea. The results showed that *Tra2a*, *Tra2*b, and *Srsf1* are expressed in both hair cells and supporting cells. A strong expression of *Tra2b* in the spiral ganglion cells was also observed (Fig. S3).

### ILDR1 affects alternative pre-mRNA splicing

**Figure 3:**
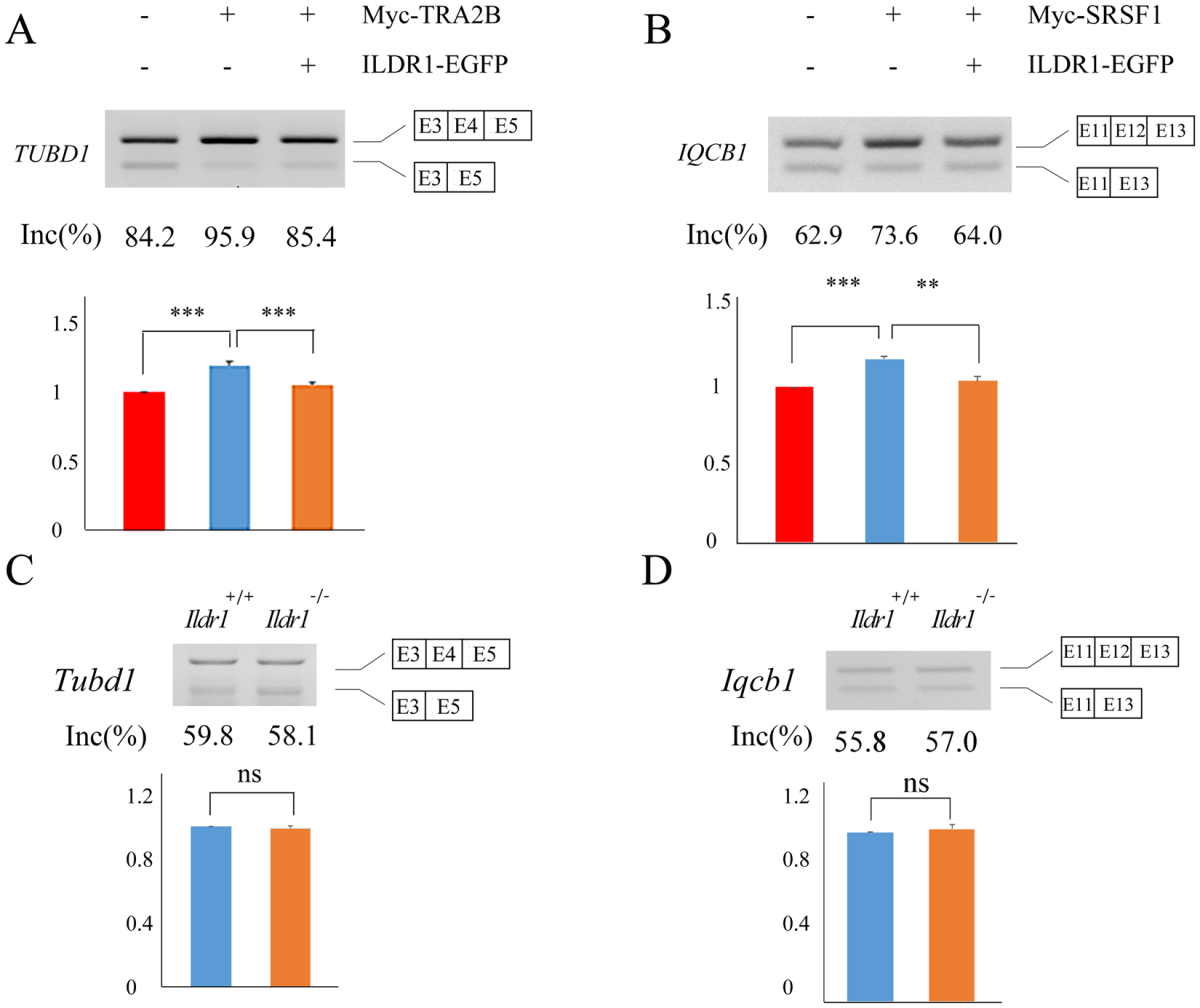
*TUBD1* and *IQCB1* pre-mRNA splicing is affected by ILDR1. (**A**) RT-PCR revealed that exon 4 of endogenous *TUBD1* in HEK293T cells is subjected to alternative splicing (lane 1). The inclusion of exon 4 is enhanced by TRA2B (lane 2), whose effect is inhibited when ILDR1 is present (lane 3). (**B**) RT-PCR revealed that exon 12 of endogenous *IQCB1* in HEK293T cell is subjected to alternative splicing (lane 1). The inclusion of exon 12 is enhanced by SRSF1 (lane 2), whose effect is inhibited when ILDR1 is present (lane 3). (**C**) The alternative splicing of *Tubd1* exon 4 was not affected in *Ildr1* knockout mice. (**D**) The alternative splicing of *Iqcb1* exon 12 was not affected in *Ildr1* knockout mice. The relative exon inclusion rate was calculated from three independently performed experiments. The differences between groups were determined by Student’s t-test. **P < 0.01; ***P < 0.001; ns, not significant.

Genome-wide analysis has revealed that TRA2A/TRA2B/SRSF1 is involved in the splicing of many alternative exons^27–29^. We picked *Tubulin delta 1* (*Tubd1*), *IQ motif containing B1* (*Iqcb1*), and *Protocadherin 19* (*Pcdh19*) as target genes to examine whether ILDR1 could affect TRA2A/TRA2B/SRSF1-mediated alternative splicing. These three genes were chosen because their alternative splicing could be readily detected in our hands. HEK293T cells were transfected with expression vectors for TRA2B (or SRSF1) with or without ILDR1, and RT-PCR results showed that TRA2B and SRSF1 promote the inclusion of exon 4 of *TUBD1* and exon 12 of *IQCB1*, respectively, whereas ILDR1 antagonizes the function of TRA2B/SRSF1 (Fig. 3A and B). As for *Pcdh19*, HEK293T cells were transfected with expression vectors for SRSF1 with or without ILDR1 alongside a minigene consisting of exon 2 and flanking exons/introns of mouse *Pcdh19* gene. RT-PCR-based evaluation of pre-mRNA splicing demonstrated that SRSF1 promotes the inclusion of *Pcdh19* exon 2, whereas ILDR1 antagonizes the function of SRSF1 (Fig. S4A). Taken together, our data suggest that ILDR1 regulates SRSF1- and TRA2B-meidated alternative pre-mRNA splicing.

We then used *Ildr1* knockout mice to examine whether loss of ILDR1 affects alternative pre-mRNA splicing in the inner ear. Total RNA were extracted from the cochlea of P0 wildtype and *Ildr1* knockout mice, and RT-PCR was performed to examine the alternative splicing of *Tubd1*, *Iqcb1*, and *Pcdh19* genes. Unexpectedly, no difference was observed between wildtype and *Ildr1* knockout mice (Fig. 3C and D, Fig. S4B). To further examine whether loss of ILDR1 affects alternative splicing, RNA from P0 cochlea of wildtype and *Ildr1* knockout mice were subjected to RNA-seq analysis, which did not reveal any significant differences in alternative gene splicing (data not shown). To verify the RNA-seq result, twenty-four genes that might show different splicing patterns according to RNA-seq result were picked and their alternative splicing in wildtype and *Ildr1* knockout mice was examined by RT-PCR. The results showed that the splicing of these genes is indeed not affected by ILDR1 deficiency (Fig. S5).

### *Ildr2* is upregulated in the inner ear of *Ildr1* knockout mice

**Figure 4:**
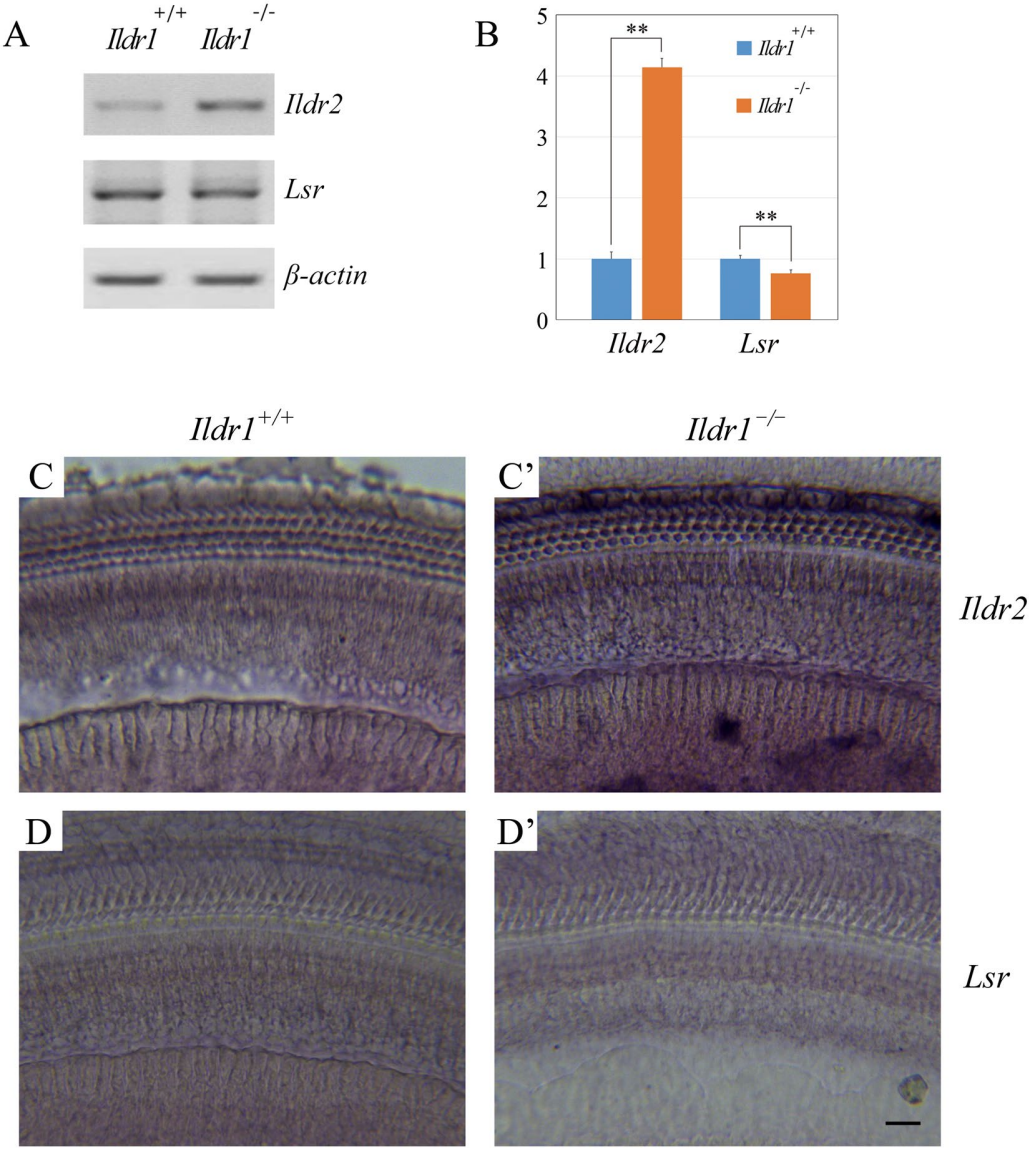
*Ildr2* is upregulated in *Ildr1* knockout mice. (**A**) RT-PCR revealed that *Ildr2*, but not *Lsr*, is upregulated in *Ildr1* knockout mice. *β-actin* was included as internal control. (**B**) Quantitative real-time PCR revealed that *Ildr2*, but not *Lsr*, is upregulated in *Ildr1* knockout mice. The differences between groups were determined by Student’s t-test. **P < 0.01. (**C**–**D’**) *In situ* hybridization showed that *Ildr2* and *Lsr* are expressed in mouse cochlea, and *Ildr2* expression is upregulated in *Ildr1* knockout mice. Scale bar: 15 μm.

The fact that alternative splicing is not affected by loss of ILDR1 prompted us to look for possible explanation. It has been shown that sometimes loss of a particular protein could be compensated for by its homologous protein. As mentioned above, ILDR1 belongs to evolutionally conserved angulin protein family, which includes ILDR1, ILDR2, and LSR. We then examined the expression of *Ildr2* and *Lsr* in *Ildr1* knockout mouse. RT-PCR and quantitative real-time PCR showed that expression of *Ildr2* is greatly increased in the basilar membrane of *Ildr1* knockout mice compared with wildtype mice (Fig. 4A and B). However, the expression of *Lsr* was not obviously affected by *Ildr1* deficiency (Fig. 4A and B). Whole-mount *in situ* hybridization was performed to examine the expression pattern of *Ildr2* and *Lsr* in the mouse cochlea. The results showed that *Ildr2* and *Lsr* are expressed in both hair cells and supporting cells (Fig. S6). *In situ* hybridization results also confirmed that *Ildr2*, but not *Lsr*, is upregulated in the cochlea in *Ildr1* knockout mice (Fig. 4C–D’).

### ILDR2 binds TRA2A/TRA2B/SRSF1

**Figure 5:**
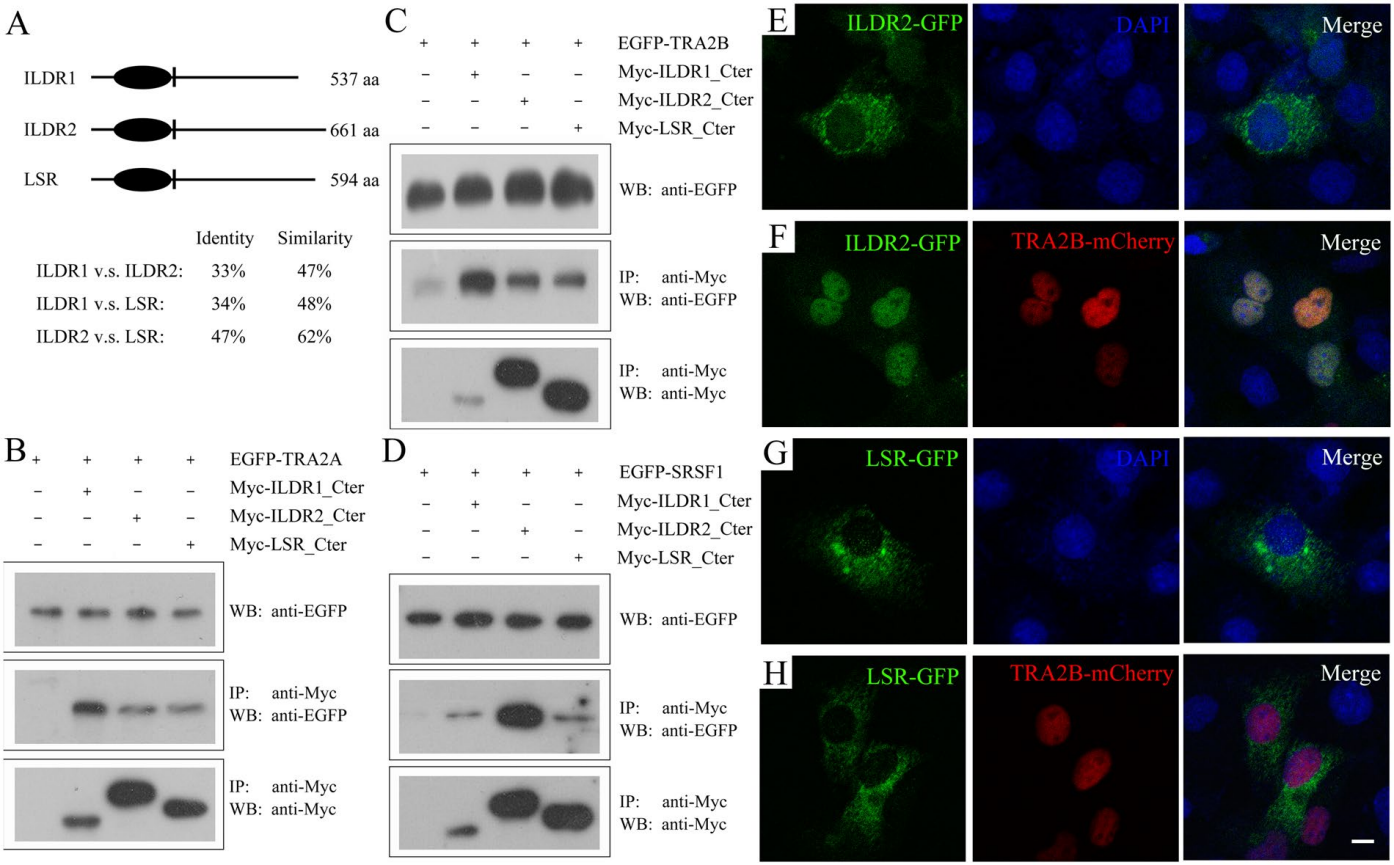
ILDR2 binds TRA2A/TRA2B/SRSF1. (**A**) Schematic drawing of the domain structures of *Mus musculus* ILDR1, ILDR2, and LSR. The identity and similarity between each other were indicated below. (**B**) Western blots showing that EGFP-tagged TRA2A was co-immunoprecipitated with Myc-tagged cytoplasmic fragment of ILDR1, ILDR2, or LSR. (**C**) Western blots showing that EGFP-tagged TRA2B was co-immunoprecipitated with Myc-tagged cytoplasmic fragment of ILDR1, ILDR2, or LSR. (**D**) Western blots showing that EGFP-tagged SRSF1 was co-immunoprecipitated with Myc-tagged cytoplasmic fragment of ILDR1, ILDR2, or LSR. Expression vectors were transfected into HEK293T cells to express epitope-tagged proteins, and cell lysis were subject to immunoprecipitation. 5% of total protein was loaded as input. IP indicates antibody used for immunoprecipitation and WB indicates antibody used for detection. Uncropped blots are shown in Fig. S10. ILDR2-GFP (**E**) and LSR-GFP (**G**) localize in the cytoplasm. However, when TRA2B-mCherry is present, ILDR2-GFP (**F**) but not LSR-GFP (**H**) translocates into the nuclei. Expression vectors were transfected into COS-7 cells to express epitope-tagged proteins. Nuclei were stained with DAPI. Scale bar: 10 μm.

We then performed experiments to examine the possibility that ILDR2 and/or LSR might regulate alternative splicing and compensate for loss of ILDR1. The three angulin proteins have similar domain architecture and share high homology between each other (Fig. 5A). First, we examined whether ILDR2 and LSR interact with TRA2A/TRA2B/SRSF1 by performing co-IP experiments. The results showed that both ILDR2 and LSR could be co-IPed together with TRA2A/TRA2B/SRSF1 (Fig. 5B–D).

Next, we examined the subcellular localization of ILDR2 and LSR in cultured cells. Similar to ILDR1, when expressed alone in cultured COS-7 cells, GFP-tagged ILDR2 and LSR localize in the cytoplasm (Fig. 5E and G). When mCherry-tagged TRA2B is present, ILDR2 translocates into the nuclei and colocalizes with TRA2B, whereas LSR still remains in the cytoplasm (Fig. 5F and H). This result suggest that although all three angulin proteins could bind the splicing factors *in vitro*, only ILDR1 and ILDR2, but not LSR, colocalize with the splicing factor in the nuclei in cultured cells.

### ILDR2 affects alternative pre-mRNA splicing

**Figure 6:**
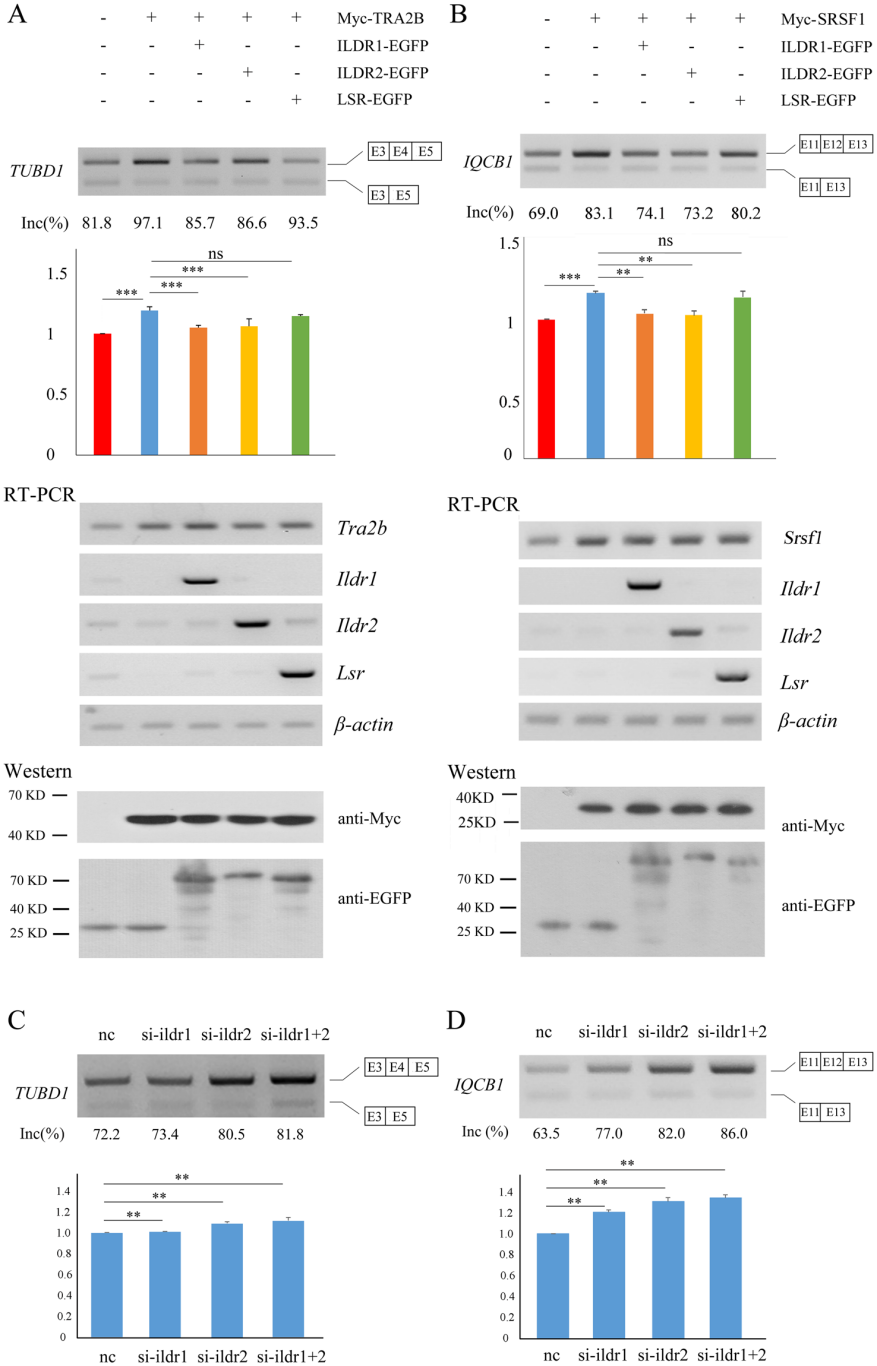
ILDR2 affects *TUBD1* and *IQCB1* pre-mRNA splicing. (**A**) RT-PCR revealed that exon 4 of endogenous *TUBD1* in HEK293T cells is subjected to alternative splicing (lane 1). The inclusion of exon 4 was enhanced by TRA2B (lane 2), whose effect was inhibited by ILDR1 (lane 3) or ILDR2 (lane 4), but not LSR (lane 5). The level of overexpression was examined via RT-PCR and Western blot. (**B**) RT-PCR revealed that exon 12 of endogenous *IQCB1* in HEK293T cells is subjected to alternative splicing (lane 1). The inclusion of exon 12 was enhanced by SRSF1 (lane 2), whose effect was inhibited by ILDR1 (lane 3) or ILDR2 (lane 4), but not LSR (lane 5). The level of overexpression was examined via RT-PCR and Western blot. (**C**) RT-PCR revealed that the inclusion of exon 4 of *TUBD1* in HEK293T cells was enhanced when ILDR1 and/or IDLR2 were knockdown. (**D**) RT-PCR revealed that the inclusion of exon 12 of *IQCB1* in HEK293T cells was enhanced when ILDR1 and/or IDLR2 were knockdown. The relative exon inclusion rate was calculated from three independently performed experiments. The differences between groups were determined by Student’s t-test. **P < 0.01; ***P < 0.001; ns, not significant.

*TUBD1*, *IQCB1*, and *Pcdh19* genes were used as target genes to examine whether ILDR2 and/or LSR could affect alternative splicing. RT-PCR-based evaluation of pre-mRNA splicing demonstrated that, similar to ILDR1, ILDR2 inhibits SRSF1- or TRA2B-mediated alternative splicing, whereas LSR does not (Fig. 6A and B, Fig. S7). Taken together, given the fact that *Ildr2* is upregulated in *Idlr1* knockout mice and that ILDR2 can regulate alternative splicing as ILDR1 does, we hypothesize that ILDR2 might compensate for the loss of ILDR1 in splicing regulation.

To further test this hypothesis, we knockdown the expression of endogenous *ILDR1* and/or *ILDR2* in cultured cells and examined its effect on alternative splicing. Transiently-transfected siRNA downregulates the expression of *ILDR1* or *ILDR2* specifically in HEK293T cells without affecting each other (Fig. S8A and B). This is in sharp contrast to what happens in *Ildr1* knockout mice. As a result, alternative splicing of *TUBD1* and *IQCB1* was affected in *ILDR1* or *ILDR2* knockdown cells (Fig. 6C and D). When the expression of both *ILDR1* and *ILDR2* was downregulated simultaneously with siRNAs, alternative splicing of *TUBD1* and *IQCB1* was affected to a greater extent (Fig. 6C and D, Fig. S8C and D). This result strongly supports the role of ILDR1/2 proteins in splicing regulation.

## Discussion

Angulin proteins are evolutionally conserved type I transmembrane proteins containing an extracellular Ig-like domain. At present three mammalian angulin proteins have been identified, namely LSR, ILDR1, and ILDR2, which are also known as angulin-1, angulin-2, and angulin-3, respectively^2^. All three angulin proteins have been shown to localize at tight junctions (TJs) and could recruit tricellulin, another important TJ component^2, 30^. In the present work we demonstrate that besides TJs regulation, angulin proteins are also involved in alternative pre-mRNA splicing through binding to specific splicing factors.

Our data show that when expressed in cultured cells, ILDR1 and ILDR2 bind to splicing factors TRA2A, TRA2B, or SRSF1, and translocate into the nuclei. Several lines of evidence suggest that the interaction and translocation are not caused by the overexpression of tagged proteins. First, SR family splicing factor SRSF5 was included in the co-IP experiment as a negative control, and the result showed that SRSF5 is not co-IPed with ILDR1 as TRA2A, TRA2B, or SRSF1 does. Presumably the interaction of RS domain with ILDR1 requires specific amino acids context that does not exist in SRSF5. Second, ILDR1α’ is an ILDR1 variant that only misses a di-leucine motif compared to the full length ILDR1. However, ILDR1α’ does not bind to TRA2A, TRA2B, or SRSF1, and does not translocate to the nuclei when the splicing factors are present. This result suggests that the di-leucine motif is necessary for the interaction. Third, LSR is an angulin protein family member and is homologous to ILDR1 and ILDR2. Although LSR is co-IPed with the splicing factors *in vitro*, it does not translocate into the nuclei when the splicing factors are present. Taken together, we believe that the interaction between ILDR1/ILDR2 and the splicing factors is specific.

It has been shown that transmembrane proteins such as receptor tyrosine kinases (RTK) and Notch receptor can translocate into the nuclei after cleavage by proteases. Through sequential cleavage by multiple proteases, a soluble cytoplasmic domain is released and translocates into the nuclei^31, 32^. We examined the molecular weight of ILDR1, ILDR2 and LSR by performing western blot, and found that ILDR1/ILDR2/LSR is not cleaved into smaller fragment when the splicing factors (SRSF1, TRA2A, or TRA2B) are present. This result suggests that the translocation of ILDR1 and ILDR2 into the nuclei is not mediated by protease cleavage.

Another hypothetical explanation suggests that after activation by ligands, the full-length transmembrane proteins are delivered from the cell surface to the endoplasmic reticulum (ER), then extracted into the cytoplasm and finally translocated into the nuclei, although the detailed mechanism remains elusive^31^. Interestingly, our group and others show that when heterogeneously expressed in cultured cells, ILDR1, ILDR2 or LSR mainly localizes in the cytoplasm with an ER-like pattern^1^. In fact, exogenous ILDR2 has been shown to primarily locate in the ER of cultured hepatoma and neuronal cells^33^. In this scenario, the splicing factors might participate in the shuttling of ILDR1/ILDR2 into the nuclei through binding to them. Further investigation is needed to fully understand the detailed mechanism.

Our data show that the interaction with angulin proteins requires the RS domain of TRA2A/TRA2B/SRSF1. RS domain is involved in protein-protein interactions that facilitate recruitment of the spliceosome^34, 35^, or directly contact the pre-mRNA to promote spliceosome assembly^36, 37^. RS domain was also suggested to act as a nuclear localization signal (NLS) and regulate the nuclear localization of SR proteins through binding to transpotin-SR^38, 39^. Here we show that the interaction of the RS domain with angulin proteins does not affect the nuclear localization of TRA2A/TRA2B/SRSF1. Nevertheless, interaction with the RS domain interferes with TRA2A/TRA2B/SRSF1-mediated alternative splicing through a yet unknown mechanism.

We do not observe any changes in alternative splicing in *Ildr1* knockout mice. There has been evidence suggesting that angulin proteins could compensate for the loss of each other. For example, in *Ildr1* knockout mice, compensatory TJ localization of LSR was observed in the organ of Corti, which is believed to be responsible for recruiting tricellulin to TJs in the absence of ILDR1^5^. In the present work, we found that *Ildr2* expression is upregulated in the inner ear of *Ildr1* knockout mice, and might compensate for ILDR1 deficiency in alternative splicing regulation. Consistently, alternative splicing is affected when endogenous ILDR1 and ILDR2 expression is knockdown in cultured cells, strongly supporting the role of ILDR1/2 proteins in splicing regulation.

In the present work, we show that ILDR1/ILDR2 could regulate the alternative splicing of *TUBD1*, *IQCB1*, and *Pcdh19*. *TUBD1* encodes delta-tubulin that is associated with the centrioles^40^. In testis, delta-tubulin localizes at the manchette in the sperm head as well as along the principal piece of sperm flagellum, and is involved in sperm maturation^41, 42^. *IQCB1* encodes an IQ domain-containing protein nephrocystin 5, and mutation of *IQCB1* gene is the most frequent cause of the renal-retinal Senior-Loken syndrome (SLSN)^43^. IQCB1 localizes to the primary cilia of renal epithelial cells and connecting cilia of photoreceptor cells, and is required for the trafficking of membrane cargos to the cilia^44^. *PCDH19* encodes a delta-protocadherin, and mutations of *PCDH19* are associated with epilepsy and mental retardation^45–47^. Transcriptome analysis suggested that *Tubd1*, *Iqcb1*, *and Pcdh19* are expressed in the mouse inner ear (SHIELD; https://shield.hms.harvard.edu)^25, 26^, whereas their exact roles in hearing remain elusive. Further investigation is also needed to identify more target genes other than *TUBD1*, *IQCB1*, and *Pcdh19* whose alternative splicing is regulated by ILDR1/2.

## Materials and Methods

### Animals

Generation and characterization of *Ildr1* knockout mice have been described previously^6^. All animal experiments were approved by the Ethics Committee of Shandong University and conducted accordingly. All methods were performed in accordance with the relevant guidelines and regulations.

### Plasmid construction

The cDNA encoding the cytoplasmic part of chicken ILDR1 (amino acids 228–553) was cloned into vector pBD-GAL4 Cam (Stratagene, La Jolla, CA, USA) to express the bait protein for yeast two-hybrid screening. The coding sequences of mouse *Ildr1*, *Ildr2*, and *Lsr* were cloned into pEGFP-N2. The coding sequences of mouse *Tra2a*, *Tra2b*, *Srsf1*, and *Srsf5* were cloned into pmCherry-N1, pEGFP-C2, and pMyc-C2 (modified pEGFP-C2 with EGFP-coding sequence replaced by Myc-coding sequence). The cDNA encoding the cytoplasmic part of mouse ILDR1, ILDR2, and LSR were cloned into pMyc-C2. The cDNA encoding the RS and RRM domains of mouse TRA2A, TRA2B, and SRSF1 were cloned into pEGFP-C2. Mouse *Pcdh19* minigene was amplified from mouse genomic DNA and cloned into pcDNA3.1(+). All the constructs were verified by Sanger sequencing.

### Yeast two-hybrid screening

Yeast two-hybrid screening was performed as described previously^48, 49^. Briefly, yeast strain AH109 (Clontech, Mountain View, CA, USA) was sequentially transformed with the bait plasmid and a chicken cochlear cDNA library in HybriZAP pAD-GAL4 vector^50^. *HIS3* was used as the reporter gene for the screening in presence of 2.5 mM 3-amino-1,2,4-triazole (3-AT). Positive colonies were further tested for activation of two other reporter genes, *ADE2* and *lacZ*. Then the pAD-GAL4 prey vectors in triple-positive colonies were recovered, and cDNA inserts were determined by Sanger sequencing.

### RNA extraction and RT-PCR

Total RNA was isolated from mouse tissues or cells transfected with expression vectors using TRIzol reagent (Ambion, Carlsbad, CA, USA) according to the manufacturer’s instructions. Reverse transcription (RT) was carried out using a cDNA synthesis kit (TaKaRa Bio Inc., Dalian, China). Polymerase chain reaction (PCR) was performed using this cDNA as template with the following primers: *Ildr1* forward primer, CCGGCGGCTGATGAAGAAAGACTC, reverse primer, AGGGCAGCAACAGCGGGTAGGA (706 bp); *Ildr2* forward primer, GGGCTGCTTGCTGATCTCTT, reverse primer, CAAAGTTCTTCCGCGACAGC (745 bp); *Lsr* forward primer, GCTATGTCAGATGTCCCTGCT, reverse primer, GTCATAGAGGTCATCCCGGC (725 bp); *Tra2b* forward primer, TTCCCGAAGTGGAAGTGCTC, reverse primer, CCTGCGATAATCTCGGCTGT (226 bp); *Srsf1* forward primer, GGACCGCCCTTCGCCTTCGTT, reverse primer, ACTCTGTTCTCGGACCGCCTGGAC (212 bp); *β-actin* forward primer, ACGGCCAGGTCATCACTATTG, reverse primer, AGGGGCCGGACTCATCGTA (372 bp). To achieve the best possible sensitivity and specificity, cycle lengths for different PCR reaction sets were adjusted between 24 and 36 cycles, and annealing temperatures were adjusted between 55 and 62 °C. The PCR products were separated by electrophoresis on agarose gel.

### Quantitative real-time PCR

Quantitative real-time PCR was carried out using SYBR® Premix Ex Taq^TM^ system (Perfect Real Time, Takara). The primers and template were the same as that used in RT-PCR. Amplification and detection were run in a Roche 480 Sequence Detection System with an initial cycle of 95 °C for 10 s followed by 40 cycles of 95 °C for 5 s, 62 °C for 10 s and 72 °C for 5 s. All PCR reactions were performed in triplicate.

### Cell culture, transfection and immunofluorescence assay

Cultured cells were maintained in Dulbecco’s modified Eagle’s medium (DMEM) supplemented with 10% fetal bovine serum (FBS), and transfected with expression vectors or siRNAs using jetPRIME transfection agent (Polyplus Transfection Inc., New York, NY, USA, Cat. No. PT-114–15) according to the manufacturer’s instructions. To visualize the localization of GFP- or mCherry-tagged proteins, transfected COS-7 cells that grown on glass coverslips were fixed with 4% PFA, then incubated with DAPI (Gen-View Scientific Inc., El Monte, CA, USA). Slides were mounted in mounting solution (50% glycerol/PBS) and imaged using a confocal microscope (LSM 700, Zeiss, Germany).

### Co-immunoprecipitation (co-IP) and western blot

HEK293T cells were transfected with expression vectors as described above, then washed twice with PBS 24 hours after transfection and resuspended in ice-cold lysis buffer containing 150 mM NaCl, 50 mM Tris at pH 7.5, 1% (vol/vol) Triton X-100, 1 mM PMSF, and 1 × protease inhibitor cocktail (Roche, Basel, Switzerland). After centrifuging at 4 °C for 20 minutes, the supernatant was collected and incubated with immobilized anti-Myc antibody (Sigma-Aldrich, St. Louis, MO, USA, Cat. No. E6654) at 4 °C overnight. Immunoprecipitated proteins were washed three times with 500 mM lysis buffer and then analyzed by western blot. Protein samples were resolved by 12% SDS-PAGE, then transferred to a PVDF membrane (Millipore, Billerica, MA, USA). After blocking in PBS containing 5% BSA and 0.1% Tween-20, the membrane was incubated with mouse monoclonal anti-Myc antibody (Sigma-Aldrich, Cat. No. M4439, 1:5000 diluted) or mouse monoclonal anti-GFP antibody (Abmart, Shanghai, China, Cat. No. M20004, 1:5000 diluted) at 4 °C over night, followed by incubation with HRP-conjugated goat anti-mouse secondary antibody (Bio-Rad, Hercules, CA, USA, Cat. No. 170–6516) at 4 °C for an hour. The signals were detected with the ECL system (Cell Signaling Technology, Danvers, MA).

### Whole-mount ***in situ*** hybridization

Templates for probe transcription were amplified by PCR and cloned into pBS(-) vector. The PCR primer sequences were as below (EcoR I and Sal I restriction sites are underlined): *Ildr2* forward primer, 5′-AATGAATTCGGAGAATCCTTGGGC-3′, reverse primer, 5′-ATTGTCGACGTACCCGGCCTTGGC-3′ (540 bp); *Lsr* forward primer, 5′-AATGAATTCCGCCGGCGGCCAGCG-3′, reverse primer 5′-ATTGTCGACCTGCGTACGCCTCGT-3′, (540 bp); *Tra2a* forward primer, 5′-AATGAATTCATGAGTGATGTAGAGGAG-3′, reverse primer 5′-ATTGTCGACTCAATAGCGTCTTGGACT-3′, (849 bp); *Tra2b* forward primer, 5′-AATGAATTCATGAGCGACAGCGGCGAG-3′, reverse primer 5′-ATTGTCGACTTAGTAGCGACGAGGTGA-3′, (867 bp); *Srsf1* forward primer, 5′-AATGAATTCATGTCGGGAGGTGGTGTG-3′, reverse primer 5′-ATTGTCGACTTAAGAAAACTGTATCCA-3′, (606 bp). The vectors were then linearized with Not I or Xho I, and antisense or sense probes were transcribed using T7 or T3 RNA polymerase and labeled with digoxigenin-11-UTP (Roche). Cochleae of P8 mice were dissected out and fixed with 4% PFA. The tissues were treated with 1 mg/ml proteinase K for 15 min, then incubated overnight at 60 °C with digoxigenin-labeled probes in hybridization solution containing 50% (v/v) formamide, 5 × saline sodium citrate, 1 mg/ml yeast RNA and 100 μg/ml heparin, followed by incubation overnight with anti-digoxigenin antibody (Roche). Signals were developed in NBT/BCIP Stock Solution (Roche) and cochleae were mounted in mounting solution (50% glycerol/PBS) and imaged with a Nikon Eclipse 800 microscope using differential interference contrast microscopy.

### RNA-seq

RNA-seq was carried out by Genesky Biotechnologies Inc, Shanghai, China. Briefly, the basilar membranes were collected from postnatal day 0 (P0) wild type or *Ildr1* ^-/-^ mice, and the RNA sequencing libraries were constructed from the extracted and amplified RNA using the standard Illumina library preparation protocols. RNA-seq was performed on Illumina HiSeq. 2000 platform using 100 bp PE protocol. Raw sequencing reads were evaluated by FastQC, then trimmed by trim_galore to remove the primers and low-quality (Q < 10) sequences. Cleaned reads were aligned to GRCm38/mm10 mouse genome assembly using Tophat with at most 2 mismatches. Alternative splicing (AS) events of samples were extracted and compared using the tool ASprofile^51^.

### Target gene splicing examination

HEK293T cells were transfected with the corresponding expression plasmids or siRNAs (Sigma-Aldrich, si-ildr1-1: SASI_Hs01_00025907; si-ildr1-2: SASI_Hs01_00025910; si-ildr2-1: SASI_Hs01_00228126; si-ildr2-2: SASI_Hs01_00228127). The efficiency of knockdown was examined by quantitative PCR with *ILDR1* forward primer 5′-CGATGTCCCCTCATCCAGTG-3′ and reverse primer 5′-GCCTCTCCACACTCCCTTTTT-3′; *ILDR2* forward primer 5′-TGTTCGCAAAGGTTACCGGA-3′ and reverse primer 5′-GCCTGAAGCTCTCTCGATCC-3′. The splicing efficiency of human *TUBD1* exon 4 was evaluated by performing RT-PCR with forward primer 5′-GGTTCTGGAAACAACTGGGC-3′ and reverse primer 5′-AGCTGATGTGCGAGGACTTG-3′. The splicing efficiency of human *IQCB1* exon 12 was evaluated by performing RT-PCR with forward primer 5′-GCTTCCATCTGCTGTGATTGC-3′ and reverse primer 5′-GAGTTCTTGGAGTCCTCGCC-3′. Splicing of mouse *Tubd1* exon 4 in wild-type or *Ildr1* knockout mice was evaluated by performing RT-PCR with forward primer 5′-GATCTGGGAACAACTGGGCA-3′ and reverse primer 5′- GTAGGCTGGAACACACTCCC-3′. Splicing of mouse *Iqcb1* exon 12 in wild-type or *Ildr1* knockout mice was evaluated by performing RT-PCR with the same primers used for human *IQCB1*. Primer sequences for examination of other genes’ splicing are listed in Table S1.

Mouse *Protocadherin 19* (*Pcdh19*) genomic sequence spanning exon 1 through 3 was PCR amplified from mouse inner ear genomic DNA using forward primer 5′-TGGAAGCTTTCCATTGCGGGCATTCTCTT-3′ containing a flanking Hind III restriction site (underlined) and reverse primer 5′-ATTGGTACCGGGAGGAGCAACTGACAACA-3′ containing a flanking Kpn I restriction site (underlined). This fragment was cloned into pcDNA3.1(+) to construct the reporter minigene plasmid, which was then used for transfection into HEK293T cells together with other expression plasmids. The splicing efficiency of *Pcdh19* minigene was evaluated by performing RT-PCR with forward primer 5′-TGGCAATCAAATGCAAGCGT-3′ and reverse primer 5′-ATGCCCATAGGAGTACTCAGC-3′.

### Statistical analysis

Data were presented as means ± SD from at least 3 independent experiments. The differences between groups were determined by Student’s t-test. *P < 0.05, **P < 0.01, ***P < 0.001.

## Electronic supplementary material

Supplementary Information
