# Supplementary material for: Angulin proteins ILDR1 and ILDR2 regulate alternative pre-mRNA splicing through binding to splicing factors TRA2A, TRA2B, or SRSF1
Source: Sci Rep. 2017 Aug 7;7:7466. doi: 10.1038/s41598-017-07530-z (PMC5547134; doi:10.1038/s41598-017-07530-z)
Supplement: Supplementary file 1 — Supplementary Information [file 41598_2017_7530_MOESM1_ESM.pdf]

Angulin proteins ILDR1 and ILDR2 regulate alternative pre-mRNA splicing through binding to splicing factors TRA2A, TRA2B, or SRSF1

Yueyue Liu[1], Hongyun Nie[1], Chengcheng Liu[1], Xiaoyan Zhai[1], Qing Sang[2], Yanfei Wang[1], Deli Shi[1,3], Lei Wang[2,*], Zhigang Xu[1,*]

[1]Shandong Provincial Key Laboratory of Animal Cells and Developmental Biology, Shandong University School of Life Sciences, Jinan, Shandong 250100, China.

[2]State Key Laboratory of Genetic Engineering and MOE Key Laboratory of Contemporary Anthropology, School of Life Sciences, Fudan University, Shanghai, 200032, China.

[3]Laboratoire de Biologie du Développement, Institut de Biologie Paris-Seine, Sorbonne Universités, Paris, France.

* Corresponding authors: Lei Wang, E-mail: wangleiwanglei@fudan.edu.cn

Tel/Fax: 86-21-54237860

Zhigang Xu, E-mail: xuzg@sdu.edu.cn

Tel/Fax: 86-531-88362647

Key words: ILDR1; ILDR2; alternative pre-mRNA splicing; RS protein; yeast two-hybrid; hearing loss

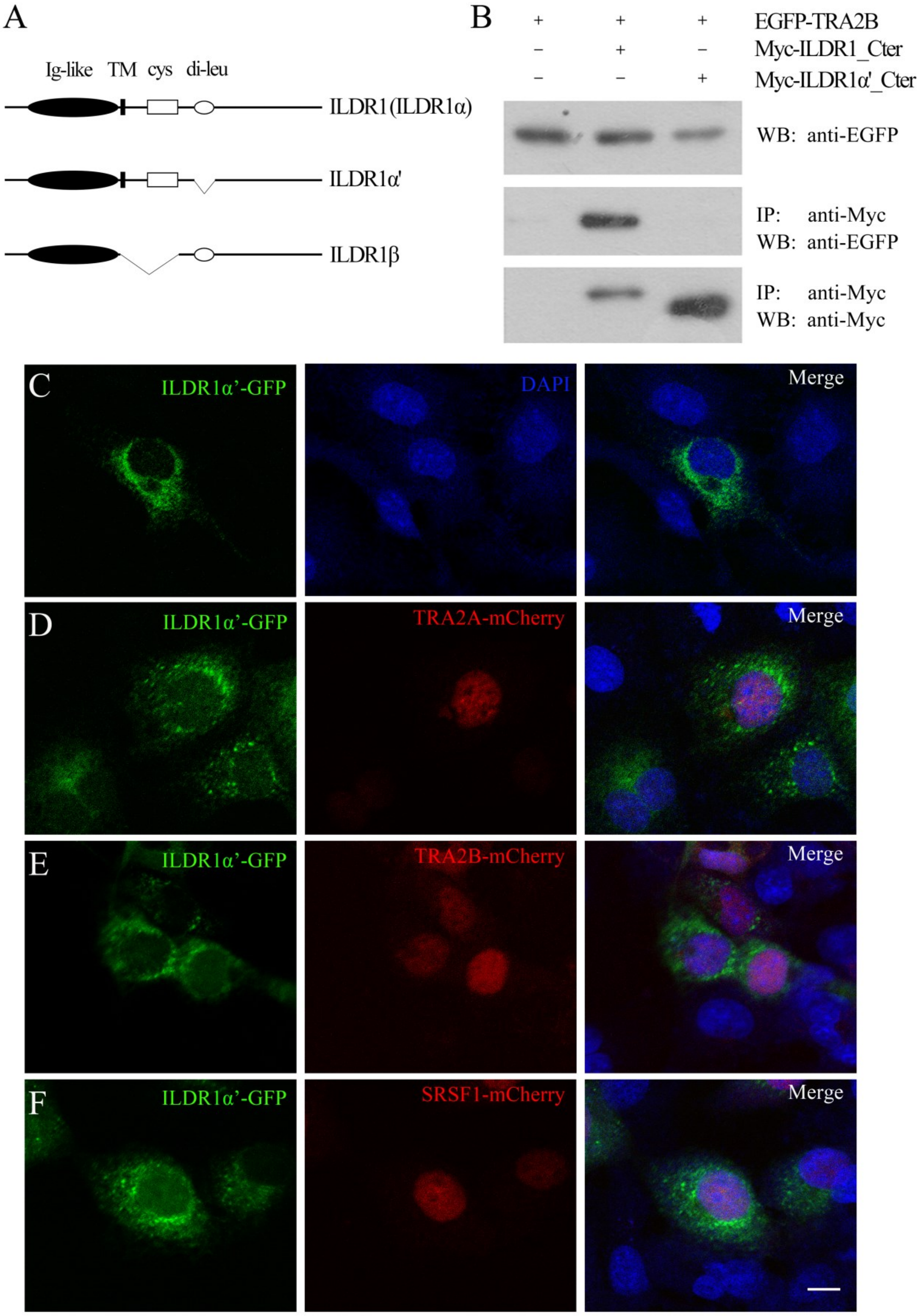

Supplemental figure S1. ILDR1α' does not interact with TRA2B. (**A**) Schematic drawing of the domain structures of three *Mus musculus* ILDR1 variants, ILDR1α, ILDR1α', and ILDR1β. (**B**) Western blots showing that EGFP-tagged TRA2B was co-immunoprecipitated with Myc-tagged ILDR1 (ILDR1α) cytoplasmic fragment, but not ILDR1α' cytoplasmic fragment. Expression

vectors were transfected into HEK293T cells to express epitope-tagged proteins, and cell lysis were subject to immunoprecipitation. IP indicates antibody used for immunoprecipitation and WB indicates antibody used for detection. (**C**) When overexpressed in COS-7 cells, ILDR1α'-GFP localizes in the cytoplasm. (**D-F**) When cotransfected with TRA2A-mCherry, TRA2B-mCherry, or SRSF1-mCherry, ILDR1α'-GFP remains in the cytoplasm, whereas the splicing factors localize in the nuclei. Expression vectors were transfected into COS-7 cells to express epitope-tagged proteins. Nuclei were stained with DAPI. Scale bar: 10 μm.

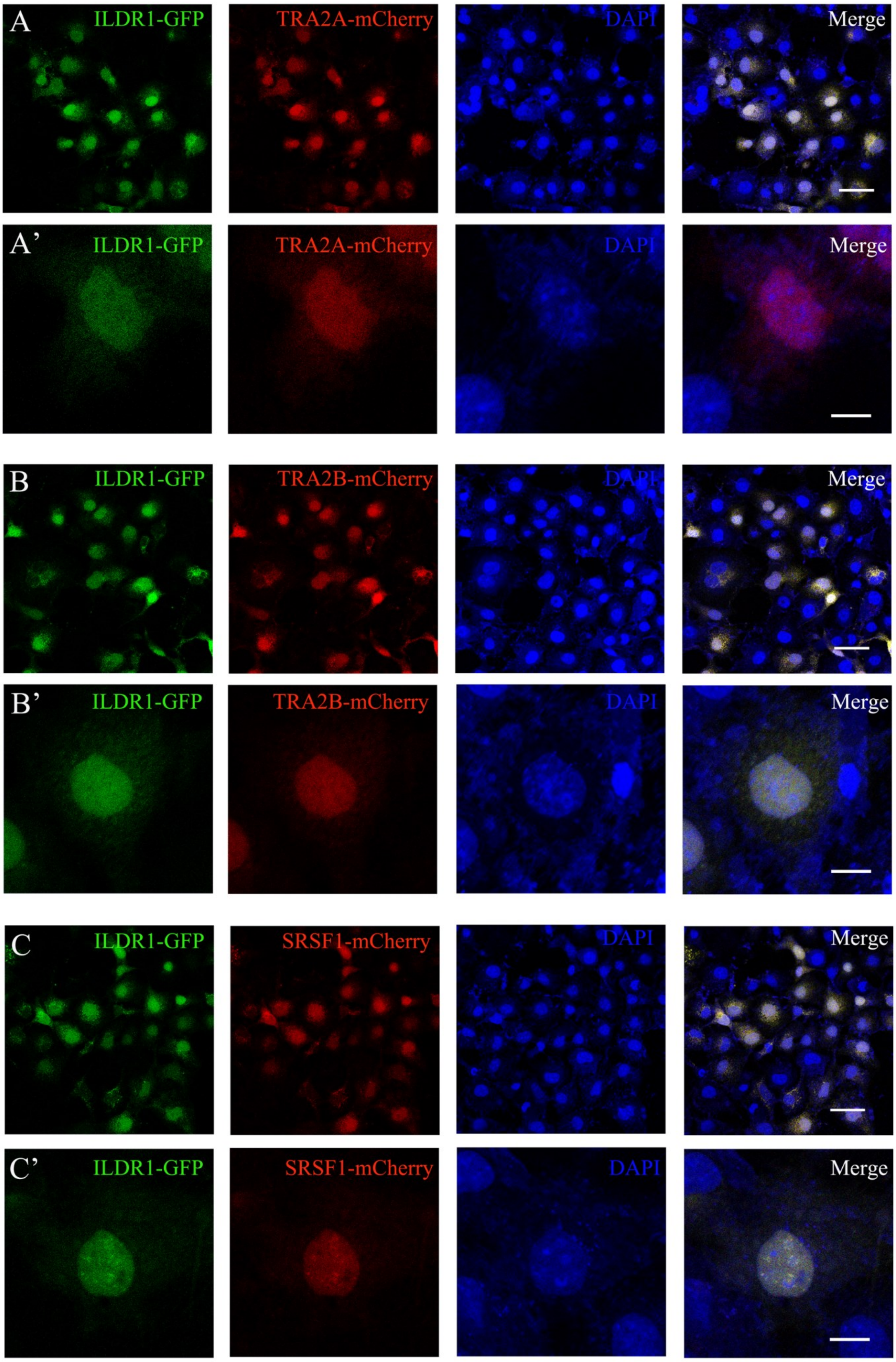

Supplemental figure S2. ILDR1 translocates into the nuclei when TRA2A, TRA2B, or SRSF1 is present. (**A**) and (**A’**) ILDR1-GFP translocates into the nuclei when TRA2A-mCherry

is present. (**B**) and (**B’**) ILDR1-GFP translocates into the nuclei when TRA2B-mCherry is present. (**C**) and (**C’**) ILDR1-GFP translocates into the nuclei when SRSF1-mCherry is present. Expression vectors were transfected into COS-7 cells to express epitope-tagged proteins. Nuclei were stained with DAPI. Scale bars: 50 µm in (A), (B) and (C), 10 µm in (A’), (B’) and (C’).

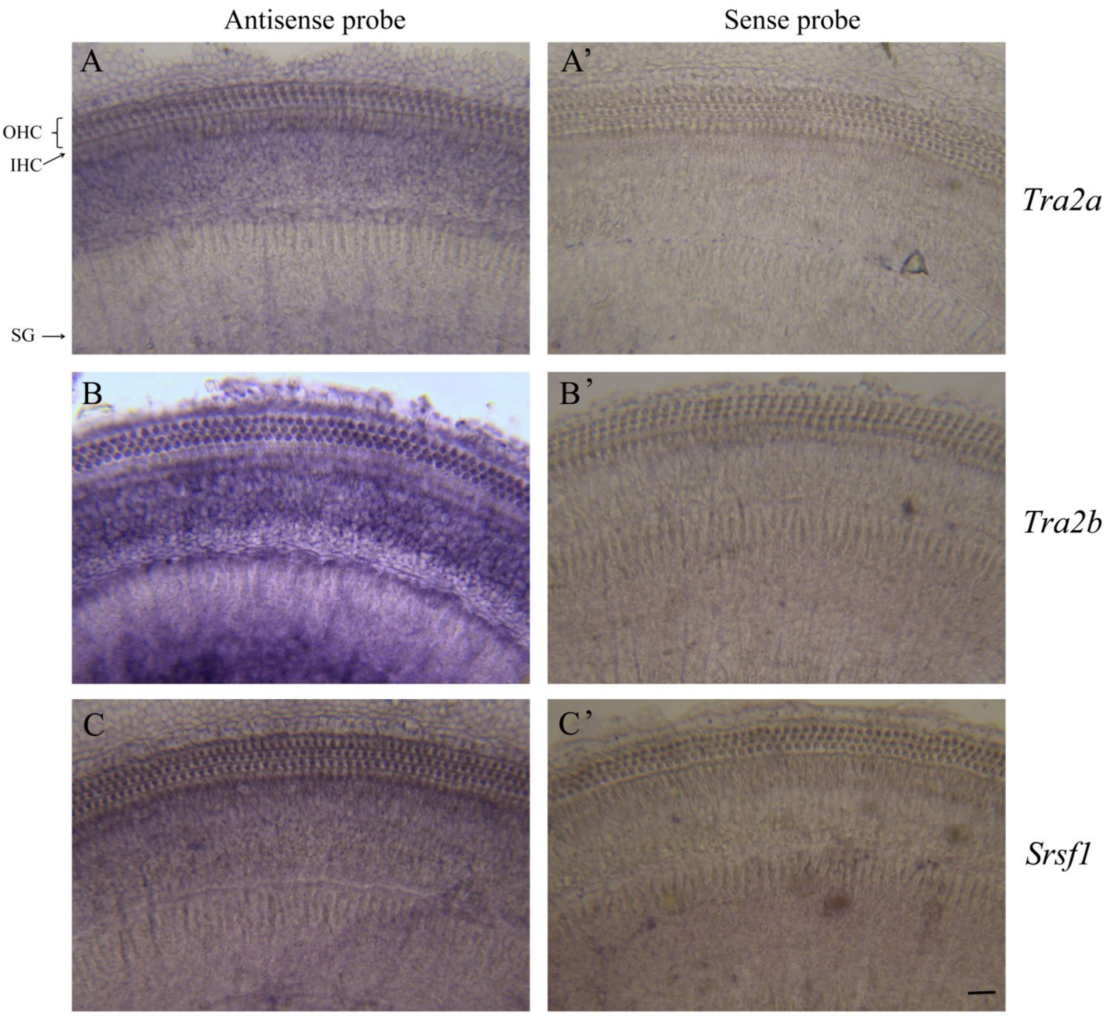

Supplemental figure S3. Expression pattern of *Tra2a*, *Tra2b*, and *Srsf1* in the mouse cochlea examined with whole-mount *in situ* hybridization. Cochlea of postnatal day 8 (P8) mice were dissected and hybridized with *Tra2a* (**A**), *Tra2b* (**B**), and *Srsf1* (**C**) antisense probes. *Tra2a* (**A'**), *Tra2b* (**B'**), and *Srsf1* (**C'**) sense probes were used as negative controls. Abbreviations: OHC, outer hair cells; IHC, inner hair cells; SG, spiral ganglion. Scale bar: 15 µm.

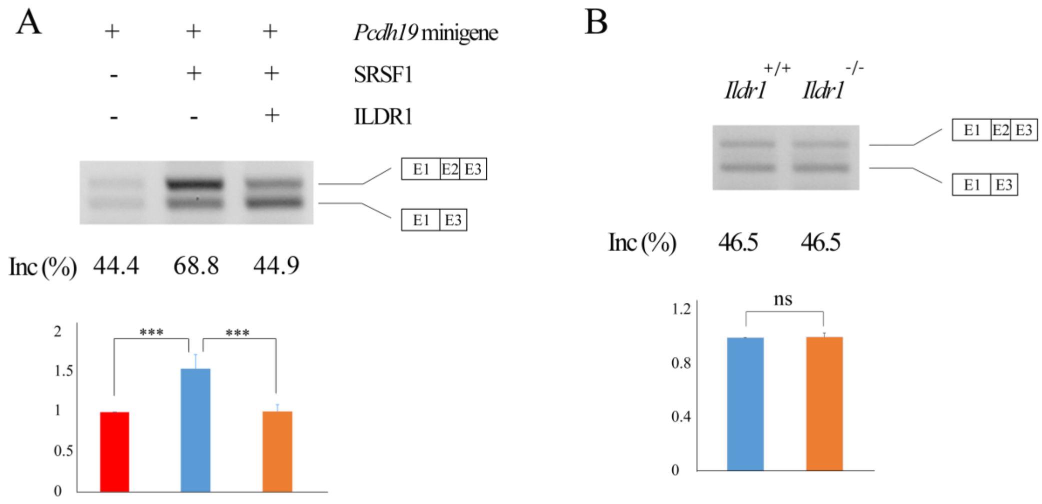

Supplemental figure S4. *Protocadherin 19* (*Pcdh19*) pre-mRNA splicing is affected by ILDR1. (**A**) RT-PCR revealed that exon 2 of *Pcdh19* minigene is subjected to alternative splicing (lane 1). The inclusion of exon 2 was enhanced by SRSF1 (lane 2), whose effect was inhibited when ILDR1 was present (lane 3). (**B**) The alternative splicing of *Pcdh19* exon 2 was not affected in *Ildr1* knockout mice. The relative exon inclusion rate was calculated from three independently performed experiments. The differences between groups were determined by Student's t-test. *** P<0.001; ns, not significant.

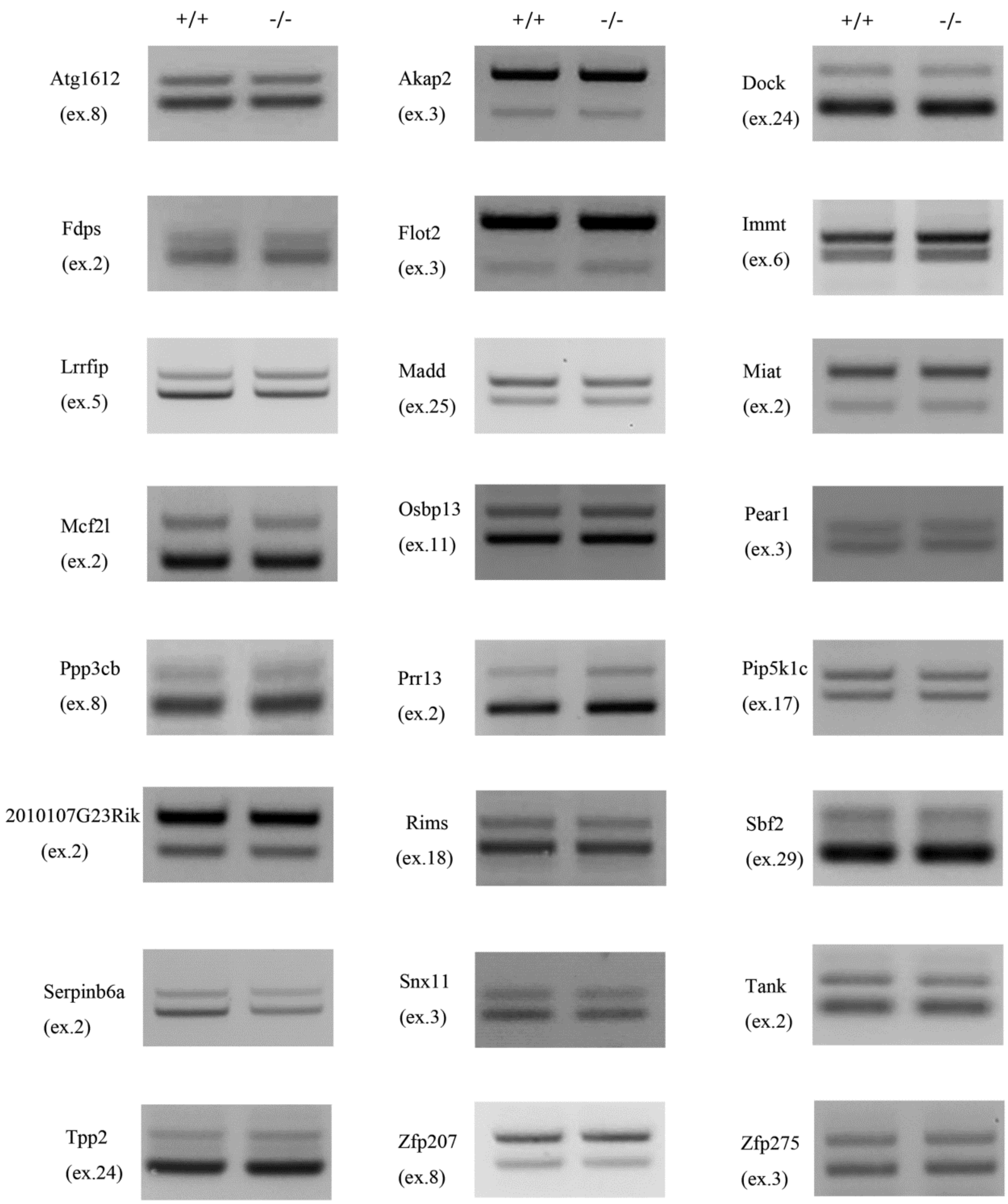

Supplemental figure S5. Pre-mRNA alternative splicing was not affected in *Ildr1* knockout mice. Total RNA of P0 mice inner ear was extracted and pre-mRNA alternative splicing of twenty-four genes was evaluated by RT-PCR.

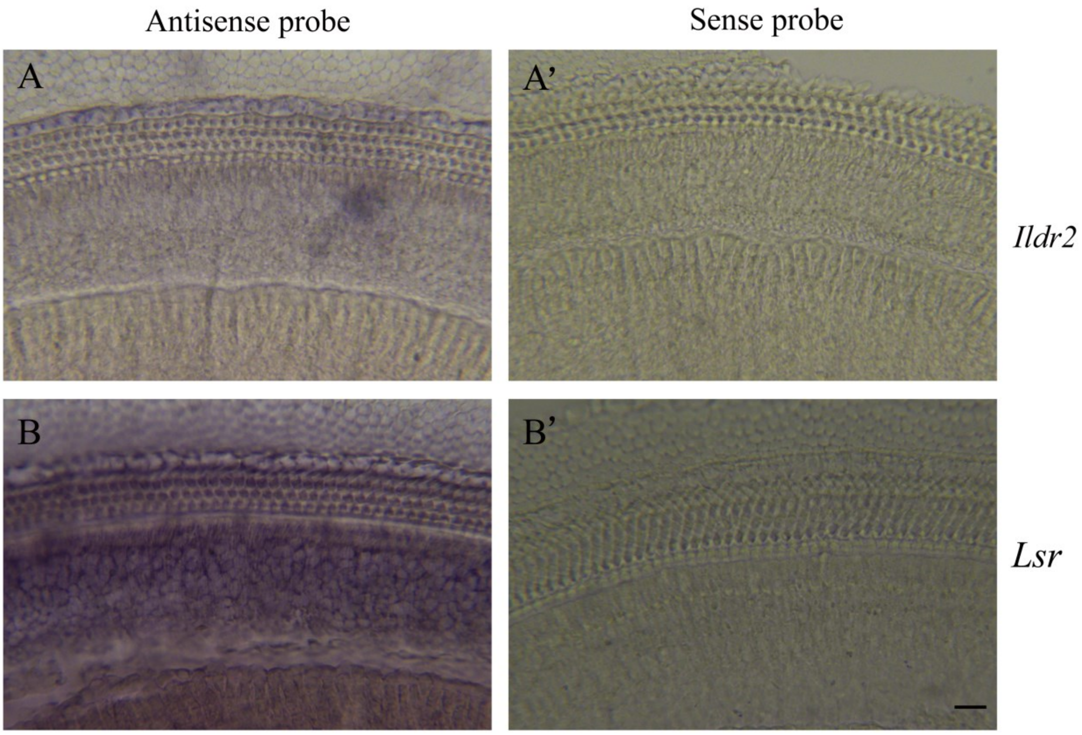

Supplemental figure S6. Expression of *Ildr2* and *Lsr* in the mouse cochlea examined with whole-mount *in situ* hybridization. Cochlea of postnatal day 8 (P8) mice were dissected and hybridized with *Ildr2* (**A**) or *Lsr* (**B**) antisense probes. *Ildr2* (**A'**) or *Lsr* (**B'**) sense probes were used as negative control. Scale bar: 15 μm.

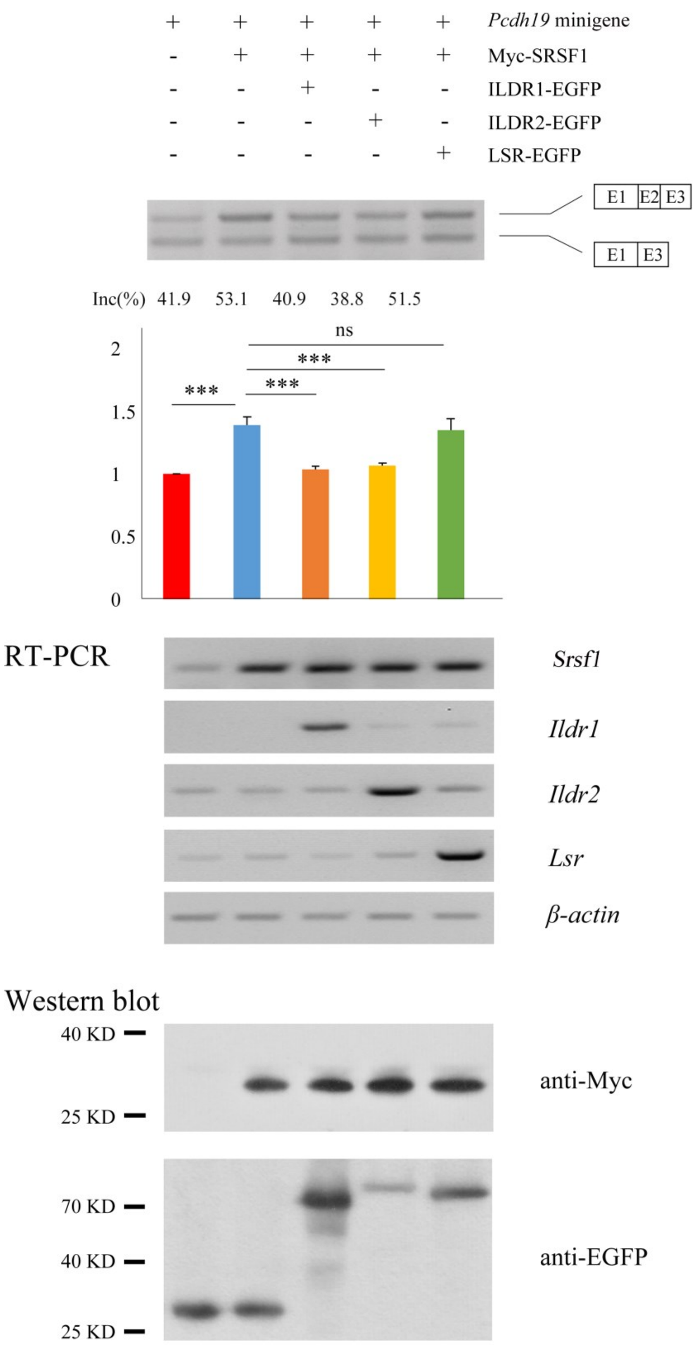

Supplemental figure S7. ILDR2 affects *Protocadherin 19* (*Pcdh19*) pre-mRNA splicing.

RT-PCR revealed that exon 2 of *Pcdh19* minigene is subjected to alternative splicing (lane 1). The inclusion of exon 2 was enhanced by SRSF1 (lane 2), whose effect was inhibited by ILDR1 (lane 3) or ILDR2 (lane 4), but not LSR (lane 5). The level of overexpression was examined via RT-PCR and Western blot. The relative exon inclusion rate was calculated from three independently performed experiments. The differences between groups were determined by Student’s t-test. *** P<0.001; ns, not significant.

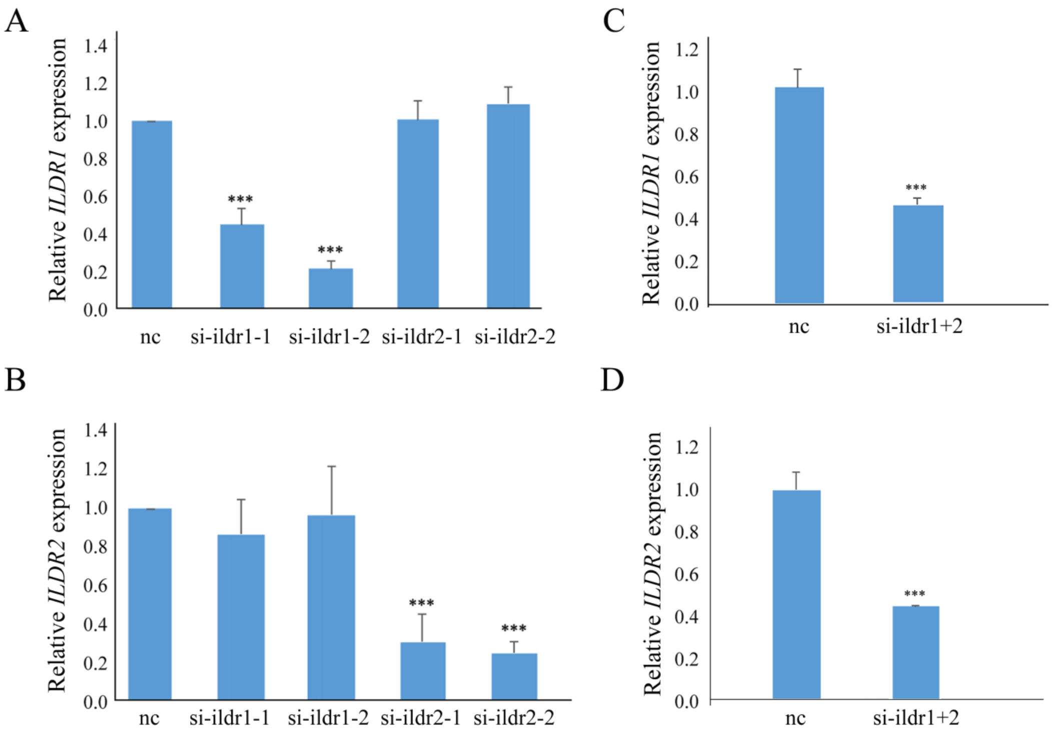

Supplemental figure S8. Examination of the siRNAs against *ILDR1* and *ILDR2* in HEK293T cells. (**A**) and (**B**) The efficiency and specificity of siRNAs against *ILDR1* and *ILDR2* was examined by quantitative PCR. (**C**) and (**D**) The efficiency of combined siRNAs (si-ildr1-2 and si-ildr2-2) was examined by quantitative PCR. The relative expression level was calculated from three independently performed experiments. The differences between groups were determined by Student's t-test. *** P<0.001.

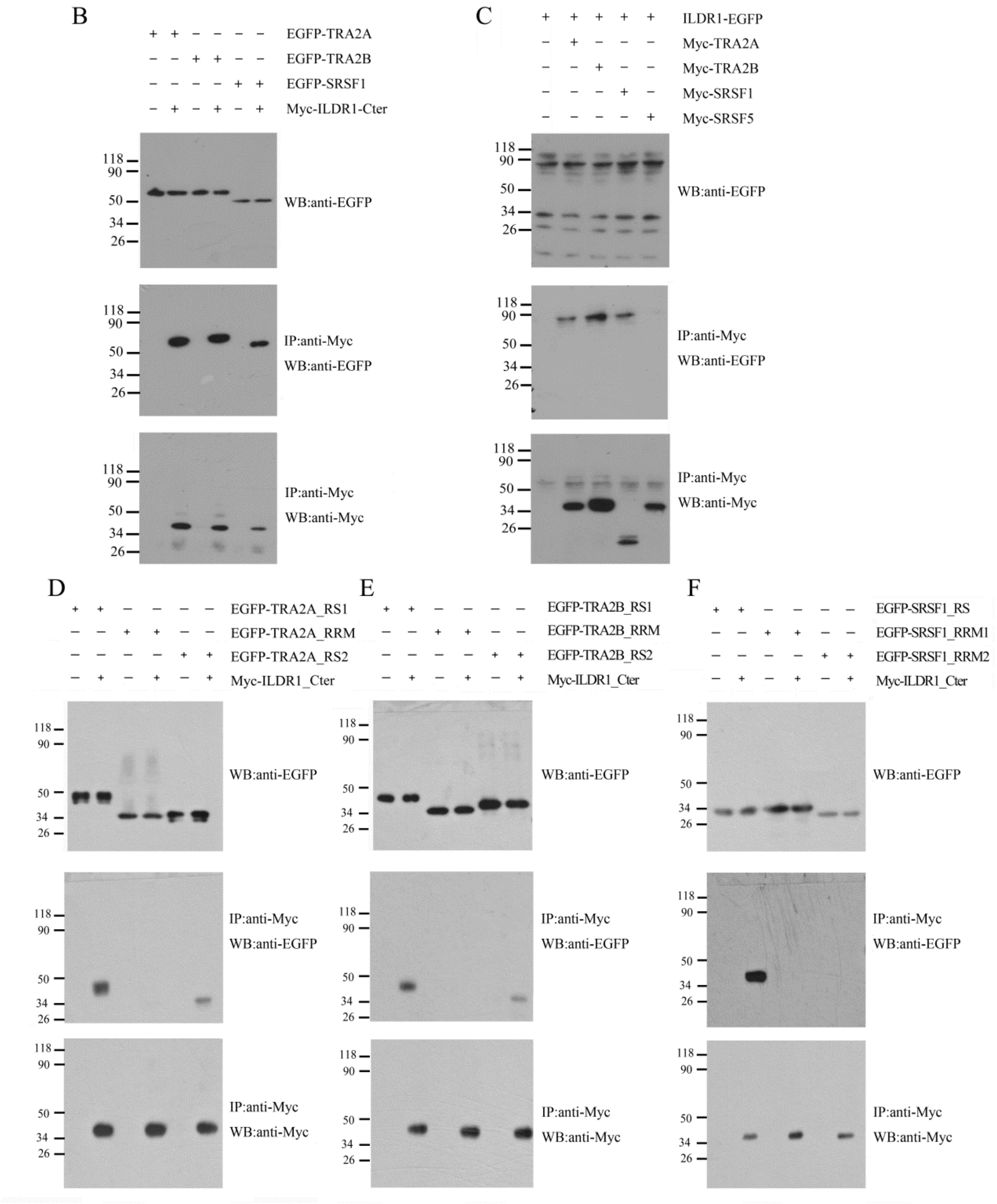

Supplemental figure S9. Uncropped blots corresponding to figure 1.

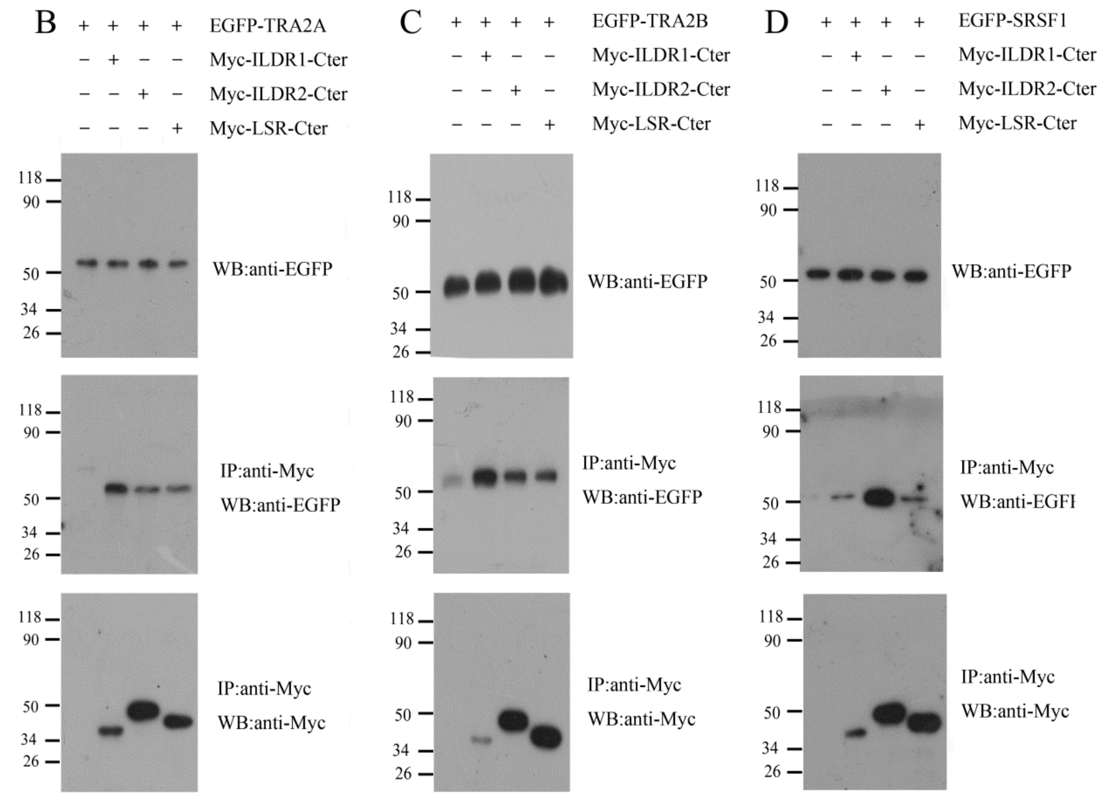

Supplemental figure S10. Uncropped blots corresponding to figure 5.

Supplementary table S1. RT-PCR primers used in splicing examination.

Gene Name	Oligonucleotide 5'>3'	product (bp)
Atg16l2-E8-F	TGTGAGCATCAGCGAGATCC	223/158
Atg16l2-E8-R	CCCAACCGAGTGACCTCTTC	
Akap2-E3-F	TCAAGGAAGCAGCGGACTTT	385/206
Akap2-E3-R	CGGAAGTCACCTTGCAAGACA	
Dock6-E24-F	CCATACTGTGGGGCTGATCC	212/119
Dock6-E24-R	AGCTGGAAGAAGAACCAGGC	
Fdps-E2-F	TGCACGCTCAGCTTTTAGGT	407/181
Fdps-E2-R	CCAGCTCCTTCTCAGTCAGC	
Flot2-E3-F	GGTCTCAGGAGGCTGTTGTG	232/141
Flot2-E3-R	GTCTCTACGTCCTCACAGCG	
Immt-E6-F	GAGACACTCCAGCTTCAGCA	302/205
Immt-E6-R	TGCAGCATTCTGGGCTGTAA	
Lrrfip1-E5-F	GCATCTCTGGGTGGGACTTC	278/206
Lrrfip1-E5-R	TCCAGCAGCATGTCCTTCAG	
Madd-E25-F	GCTACAAGCGCCATCTTTGG	284/220
Madd-E25-R	CCCTGGTCCATACCCATTCC	
Miat-E2-F	GAGGACTCCCCCAGTTCTGT	236/188
Miat-E2-R	TTGTGCCAAAGCAGTGACTC	
Mcf2l-E26-F	GAAGGCTACGTGAGCTCATCG	239/123
Mcf2l-E26-R	GAGTGGGATGTTTTGCTCCAG	
Osbpl3-E11-F	TACTTCGCTTTAAGGTCGGC	347/238
Osbpl3-E11-R	AGCAGAACTTCCTGGGCATC	
Pear1-E3-F	CCTGAAGGCCGTCGGAAG	178/104
Pear1-E3-R	GGGACAAAGTGGCATTGCAG	
Ppp3cb-E12-F	GGAGTGTTGGCTGGAGGACG	193/95
Ppp3cb-E12-R	ATGTGGTGGAGAGAATCCTCG	

Prr13-E2-F	GTGCGAACCCAGACTGAGAA	269/151
Prr13-E2-R	GTGGACAGGCAGGATTGGAA	
Pip5k1c-E17-F	CTGCCAGCCAGGCCTCAG	372/294
Pip5k1c-E17-R	AATCTTGCTGCCCCAACAGG	
2010107G23Rik-E2-F	CCCGGGAACCGTGCG	217/138
2010107G23Rik-E2-R	TGCTACTTCTGTGCTGGGTG	
Rims-E18-F	GCCGAATGGATAGACACCGT	333/153
Rims-E18-R	TTGTCTGGACAGACCCGGTA	
Sbf2-E29-F	TCTCACTGTCAGACCAGCAC	261/166
Sbf2-E29-R	GCGGCTTGTCGTTTCAAGAG	
Serpinb6a-E2-F	TCAGACTCCTCCCGACTCTC	432/264
Serpinb6a-E2-R	CCCCCATGAAGACCATAGCC	
SNX11-E3-F	TGGACGGGACAGGTTTTCTG	215/155
SNX11-E3-R	AGGAGCCTTCATTCTGCACC	
Tank-E2-F	AGCTACTTCCGGTTGCAGTC	398/193
Tank-E2-R	TATCCATGCATGCCTGTCGG	
TPP2-E24-F	CCTTGCAGGCTCCTTGACAT	221/183
TPP2-E24-R	TGAGATCGCGTAATGCTTCAGT	
Zfp207-E8-F	TCTTTTCCCCAGTGCTGGAC	285/192
Zfp207-E8-R	ATTGAAGCTGCTGGCTTTGC	
Zfp275-E3-F	CGGGTGAGACCTTCCCTACA	247/155
Zfp275-E3-R	GCTCCGGGTTCAAACTGAGA	
